# Supplementary material for: Nonvolatile ferroelectric domain wall memory integrated on silicon
Source: Nat Commun. 2022 Jul 26;13:4332. doi: 10.1038/s41467-022-31763-w (PMC9325887; doi:10.1038/s41467-022-31763-w)
Supplement: Supplementary file 1 — Supplementary Information [file 41467_2022_31763_MOESM1_ESM.pdf]

Supplementary Information

Nonvolatile ferroelectric domain wall memory integrated on silicon

Haoying Sun[1,2], Jierong Wang[1,2], Yushu Wang[1,2], Changqing Guo[3], Jiahui Gu[1,2], Wei Mao[1,2], Jiangfeng Yang[1,2], Yuwei Liu[1,2], Tingting Zhang[1,2], Tianyi Gao[1,2], Hanyu Fu[1,2], Tingjun Zhang[1,2], Yufeng Hao[1,2], Zhengbin Gu[1,2], Peng Wang[1,2,4], Houbing Huang[3], and Yuefeng Nie[1,2]*

Affiliations

[1] National Laboratory of Solid State Microstructures, Jiangsu Key Laboratory of Artificial Functional Materials, College of Engineering and Applied Sciences, Nanjing University, Nanjing 210093, P. R. China

[2] Collaborative Innovation Center of Advanced Microstructures, Nanjing University, Nanjing 210093, P. R. China

[3] School of Materials Science and Engineering & Advanced Research Institute of Multidisciplinary Science, Beijing Institute of Technology, Beijing 100081, China

[4] Department of Physics, University of Warwick, Coventry CV4 7AL, UK

These authors contributed equally: Haoying Sun, Jierong Wang

Authors to whom correspondence should be addressed: ynie@nju.edu.cn

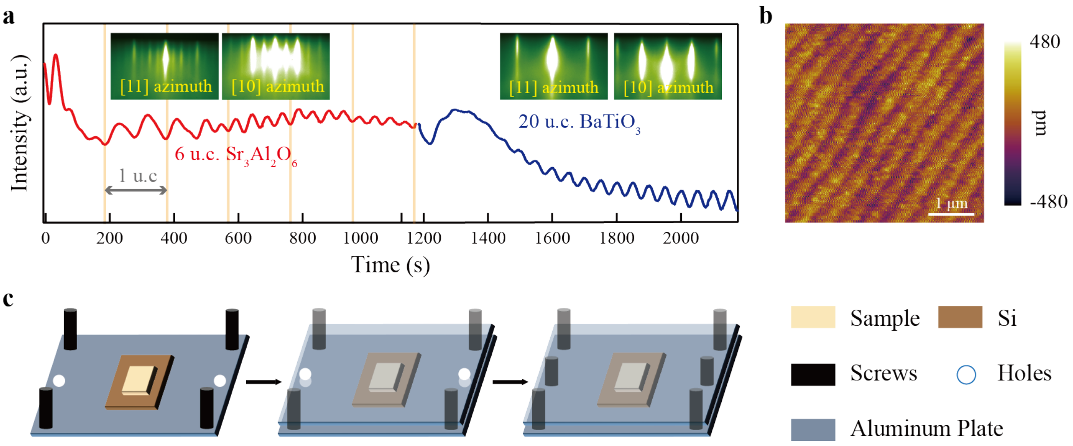

Supplementary Fig. 1 | Freestanding $BaTiO_3$ (BTO) films preparation. a, RHEED oscillation curves for 20 u.c. BTO thin film grown on STO substrate with 6 u.c. SAO sacrificial layer. Insets are corresponding RHEED diffraction patterns. Yellow vertical line indicates the growth of one unit-cell SAO layer. **b**, AFM image for as-grown 20 u.c. BTO/SAO/STO heterostructure showing clear steps and terraces. **c**, Schematics of clamping device used for fixing the heterostructure and Si substrate during transferring process.

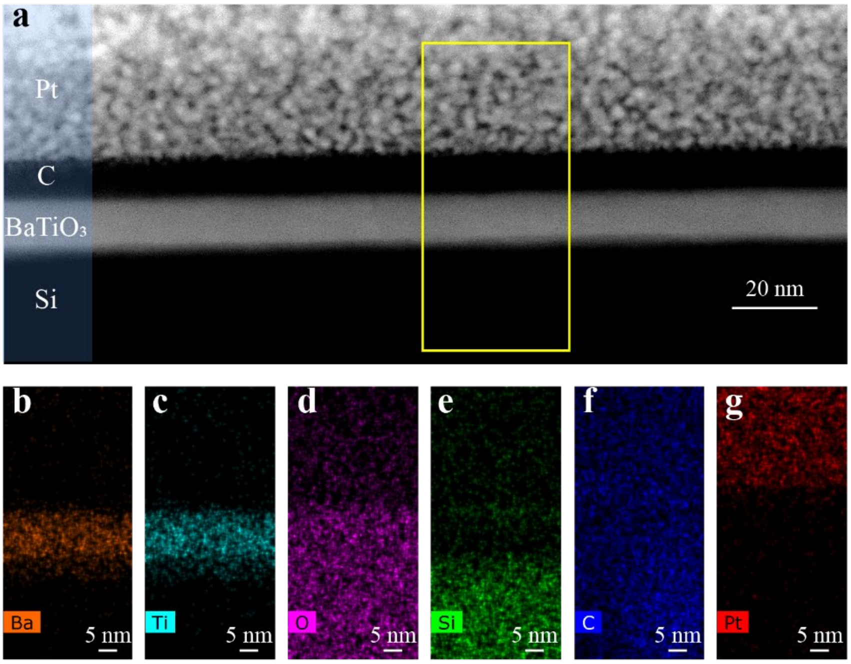

Supplementary Fig. 2 | HAADF-STEM and EDS measurements of a BTO/Si sample. a, HADDF-STEM image of Pt/C(conductive protection layer)/BTO/Si cross-sectional sample, revealing a good contact between BTO membrane and Si substrate. **b-g,** Corresponding EDS elemental maps showing the spatial distribution of Ba, Ti, O, Si, C and Pt. Note that the distribution of O in Si substrate is due to the oxidization during the TEM sample preparation and storage.

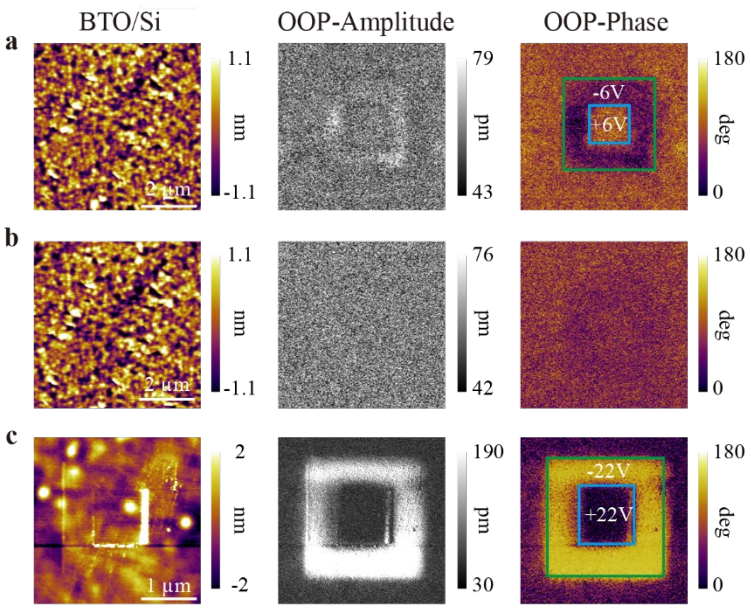

Supplementary Fig. 3 | Thickness-dependent stability variations of out-of-plane polarization in BTO membranes on Si. a,b, VPFM results for freestanding 20 u.c. BTO/Si immediately after domain switching (**a**) and an hour after domain switching (**b**). Faint phase contrast between upward (negative-biased region) and downward (positive-biased region) polarization appears instantly after switching in **a** but fades away quickly in **b**. **c**, VPFM results for freestanding 160 u.c. BTO/Si after domain switching. The results sustain for more than one hour. Note that the applied bias voltages are both above their coercive field.

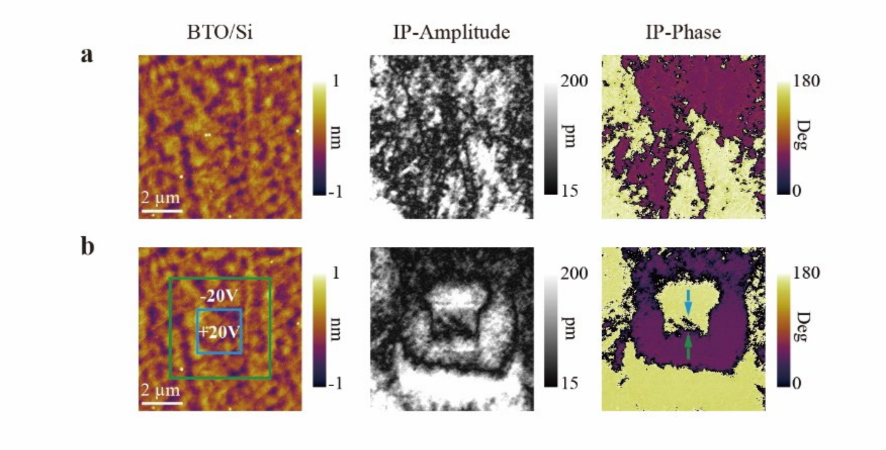

Supplementary Fig. 4 | In-plane domain switching for 160 u.c. BTO membrane on Si. a, The initial in-plane multidomain state. **b**, The switched domain state after applied bias voltages of ±20V using trailing field method. The switched domains deviate from the poled regions, due to that the in-plane polarization is actually switched by the trailing field of the biased tip.

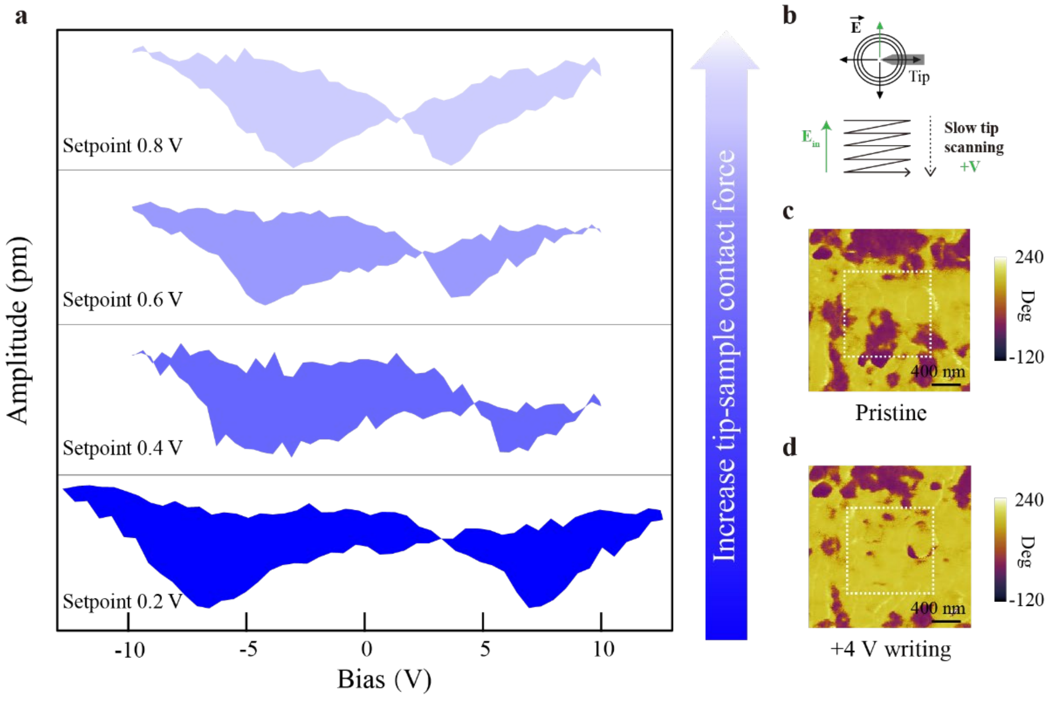

Supplementary Fig. 5 | Lower switching voltage by higher deflection setpoints. a, Butterfly-shape amplitude loops at different deflection setpoints. Coercive fields (voltages) drop with higher deflection setpoints (better tip-surface contact). **b,** Trailing field method for domain switching. **c,d,** Domain switching results at 4 V by applying higher deflection setpoint.

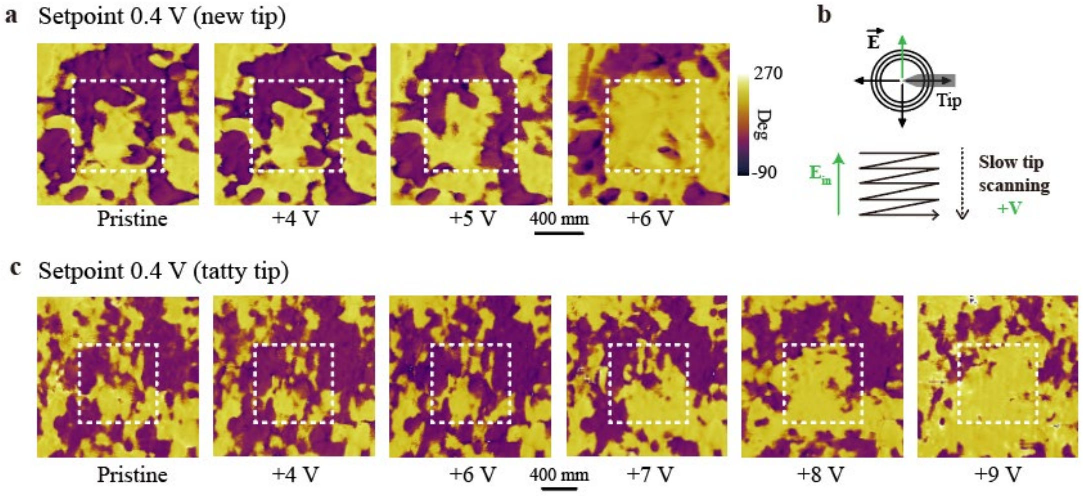

Supplementary Fig. 6 | Domain switching results by using different tips. a, Domain can be switched when switching voltage reaches +6 V by using new tip. **b,** Trailing field method of domain switching. **c,** Domain can be completely switched until switching voltage reaches +9 V by using old tatty tip.

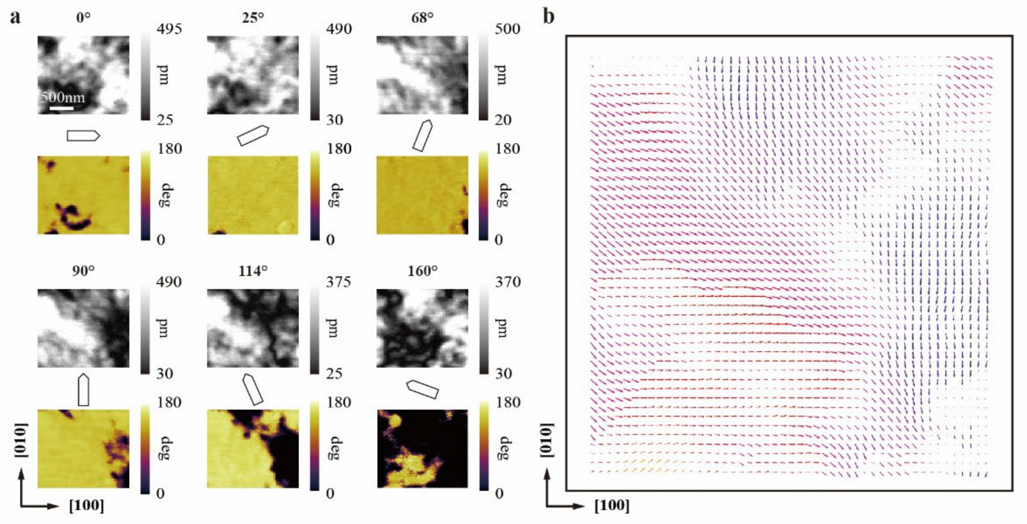

Supplementary Fig. 7 | Construction of an in-plane piezoresponse vector map. a, LPFM amplitude (upper image) and phase (lower image) results for freestanding 20 u.c. BTO/Si measured at six different rotating angles in the same region. Angle values denote the angles between the cantilever and the BTO [100] direction during LPFM scanning. **b**, Polarization mapping result for this region by fitting the data obtained in **a**. Distinct *a*-axis and *b*-axis polarization are displayed in **b**, with the lengths of the arrows representing the amplitudes of the polarization.

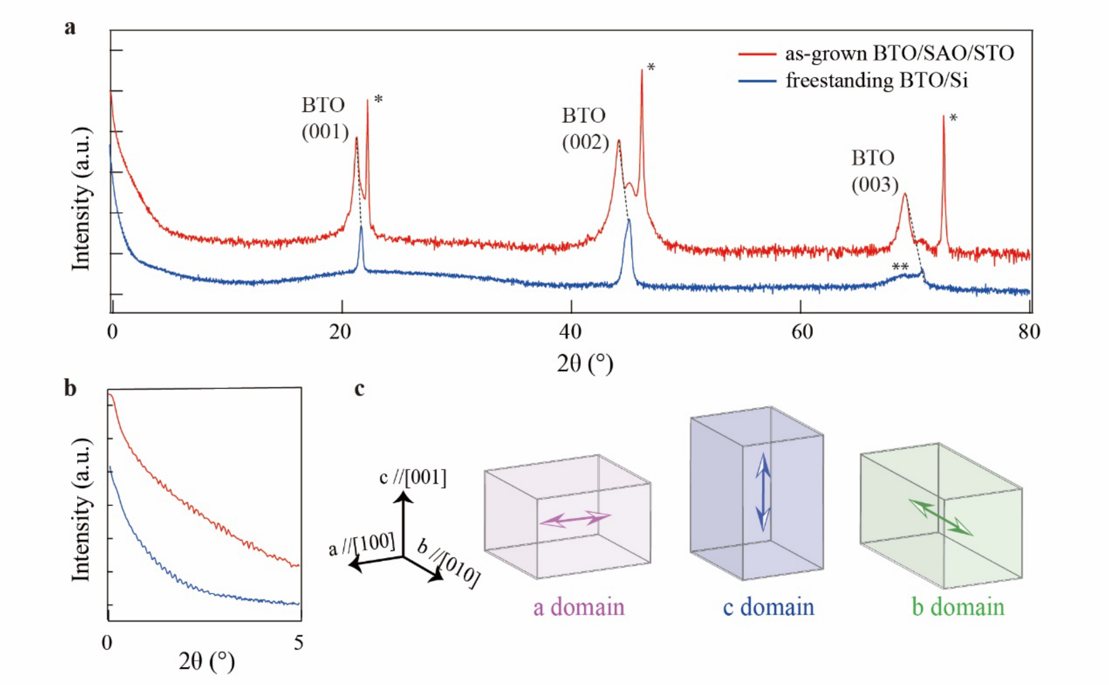

Supplementary Fig. 8 | XRD measurements for 160 u.c. BTO before and after transferring.

a, *2θ-ω* scans for 160 u.c. BTO before and after transferring. Asterisks (*) denote STO substrate diffraction peaks. Double-asterisk (**) denotes Si (004) diffraction peak of BTO/Si. **b**, Obvious Kiessig fringes at low angles for 160 u.c. BTO before and after transferring. **c**, Schematics of structural composition of BTO membrane on Si, illustrating the mixed *a*-axis, *b*-axis and *c*-axis tetragonal phases.

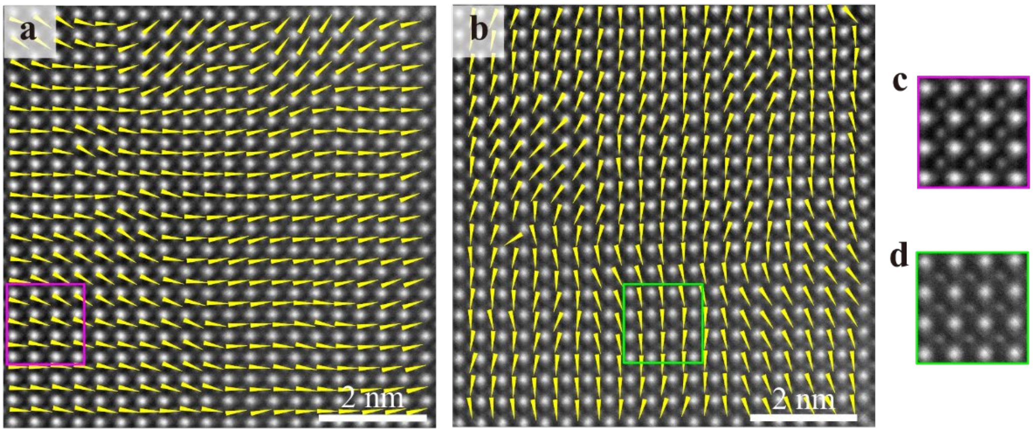

Supplementary Fig. 9 | In-plane ferroelectricity of BTO membrane. a,b, Maps of polar atomic displacement vectors obtained from the plan-view HAADF-STEM images of a 160 u.c. BTO membrane transferred to a holey carbon TEM grid. **c,d,** Zoom-in atomically resolved HAADF-STEM images. The average displacement of Ti atom is about 21 pm for ferroelectric domain in **a** and 17 pm for domain in **b**.

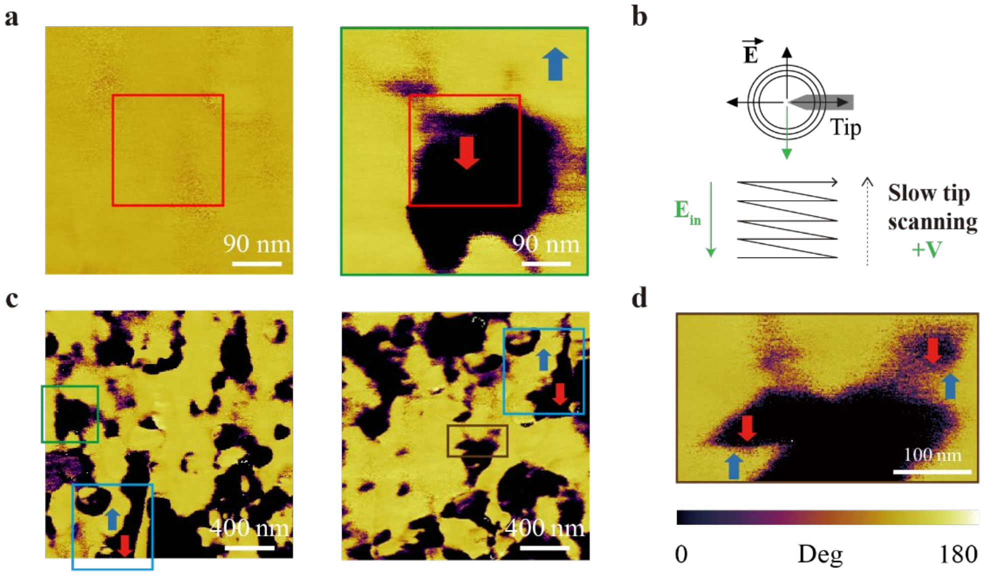

Supplementary Fig. 10 | Polarization direction determination of the region shown in Fig. 4c-e. a, The test region before (left) and after (right) domain writing. The center red box is written to be red-arrow polarized by trailing field. **b,** Schematics of trailing field method for domain switching. **c,** Based on the information in the green box (the whole test region is marked as green in the right panel of **a**), black phase-color represents red-arrow polarization and yellow phase-color represents blue-arrow polarization. The blue box ensures the consistency of left panel area and right panel area in **c**. The target region is marked as brown in right panel of **c**. **d,** Polarization result of the target region shown in Fig. 4c-e based on results in **c**.

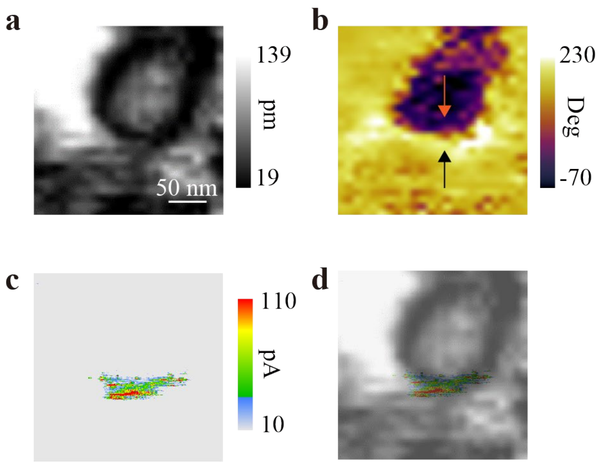

Supplementary Fig. 11 | Conductivity of intrinsic circular DW on 20 u.c. BTO/Si. a,b, LPFM amplitude (**a**) and phase (**b**) images. Arrows demonstrate polarization orientations. **c,** c-AFM current image. **d,** Overlay of amplitude image and current image showing that H-H DW where DW is nearly perpendicular to polarization exhibits conductivity.

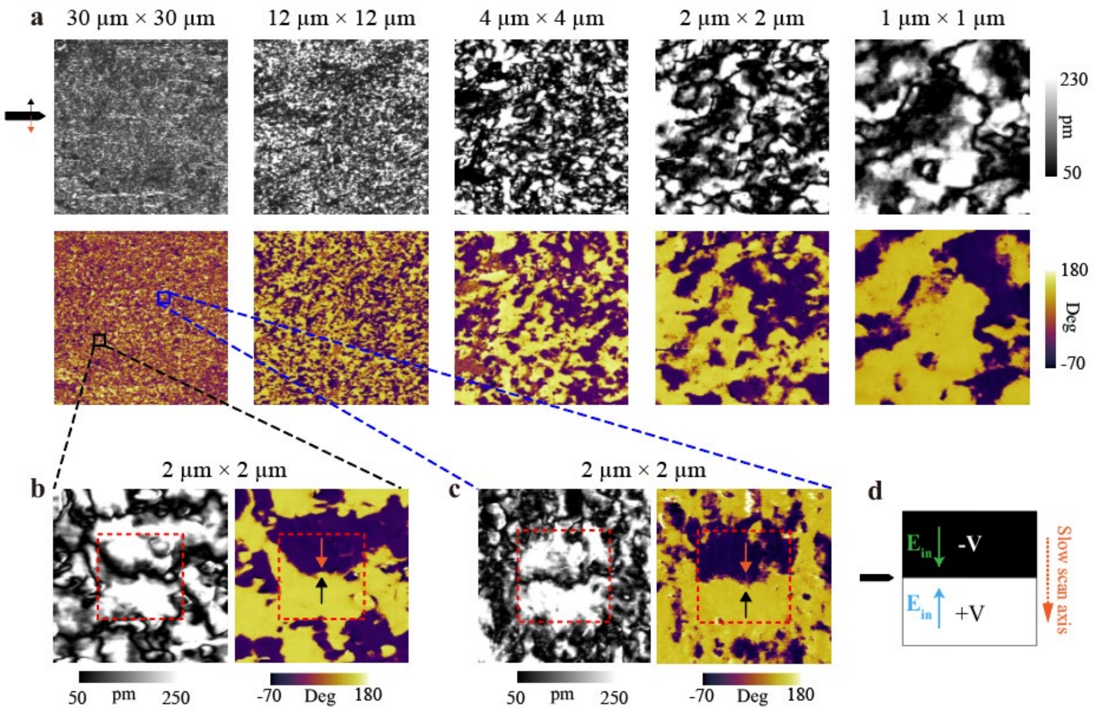

Supplementary Fig. 12 | Scale of H-H DWs. a, LPFM results from large to small area, showing that in-plane ferroelectricity is exhibited throughout the whole sample. **b,c,** Large size H-H DW created on two random regions, showing that large size H-H DW can be created on BTO/Si in both large amount and large size. **d,** Scanning method for creating H-H DWs using trailing field.

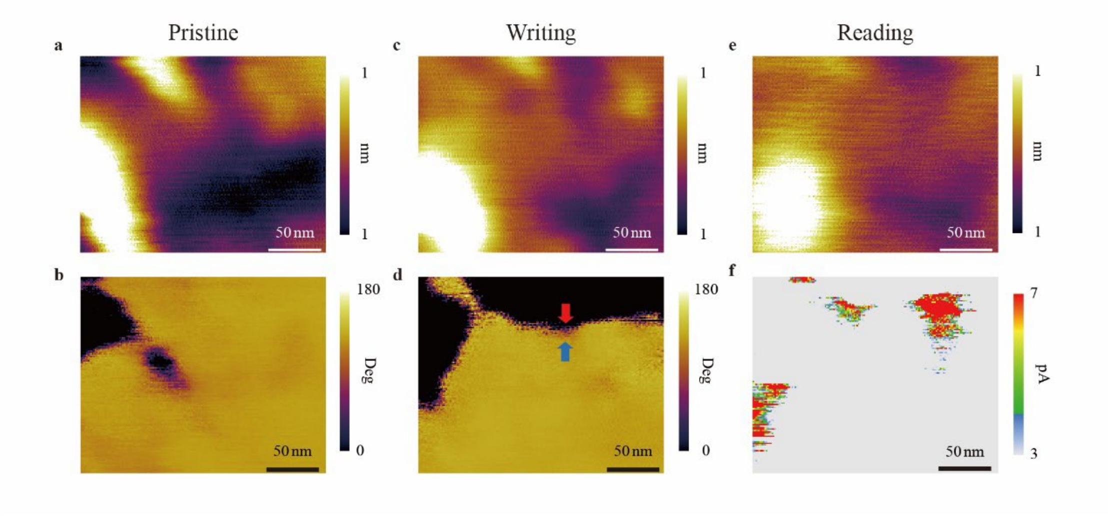

Supplementary Fig. 13 | 'Writing' and 'reading' H-H DW on 20 u.c. BTO/Si. a,b, AFM height image (**a**) and LPFM phase image (**b**) before 'writing'. **c,d,** AFM height image (**c**) and LPFM phase image (**d**) after 'writing'. **e,f,** AFM height image (**e**) and c-AFM current image (**f**) by 'reading' process.

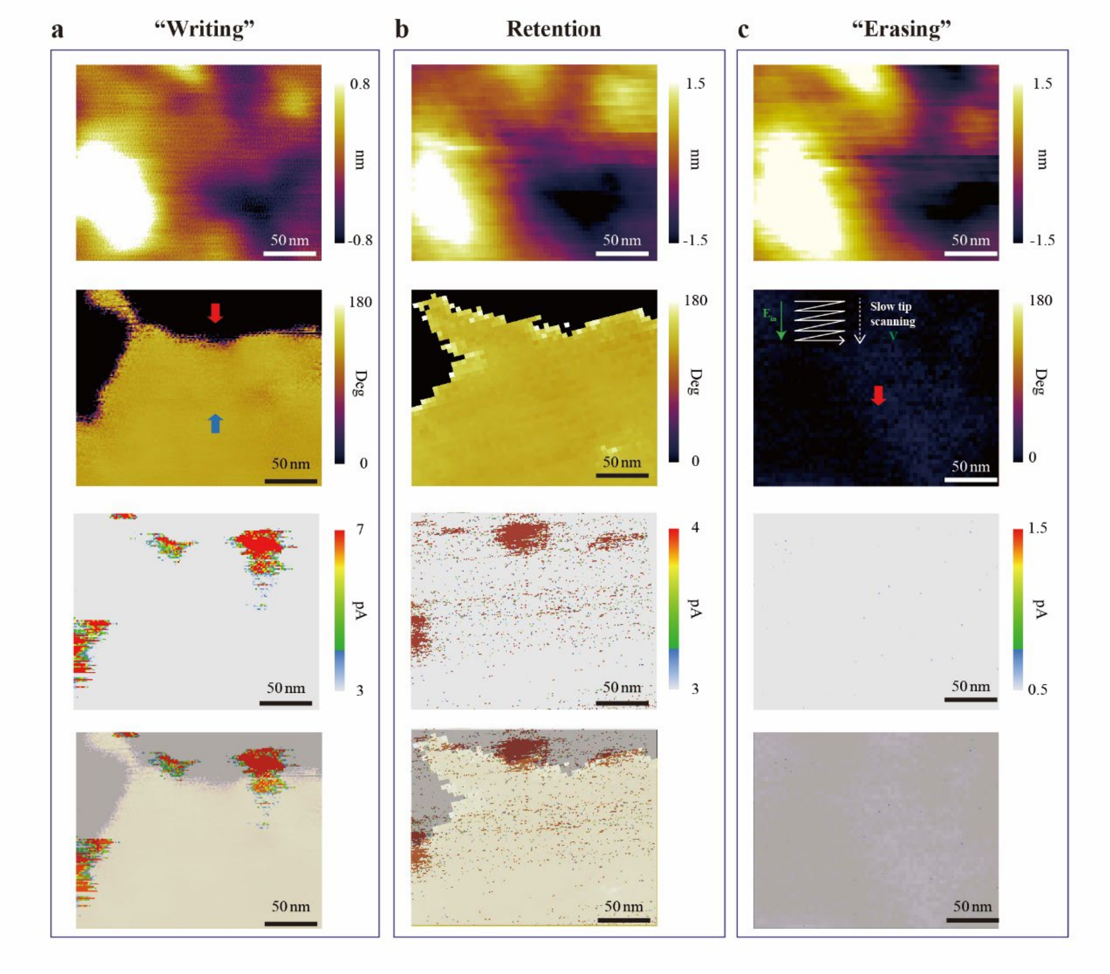

Supplementary Fig. 14 | Retention and erasure of the conductive DWs on 20 u.c. BTO/Si.

a, The written conductive DWs on 20 u.c. BTO/Si. **b,** scanning of the created conductive DWs 8 months after creating it. Reading voltage is at +3 V. **c** Erasing the created conductive DW by scanning over the whole region with the same negative voltage. From top to bottom: height image, LPFM phase image (arrows for polarization directions), c-AFM current image and phase-current overlapping image.

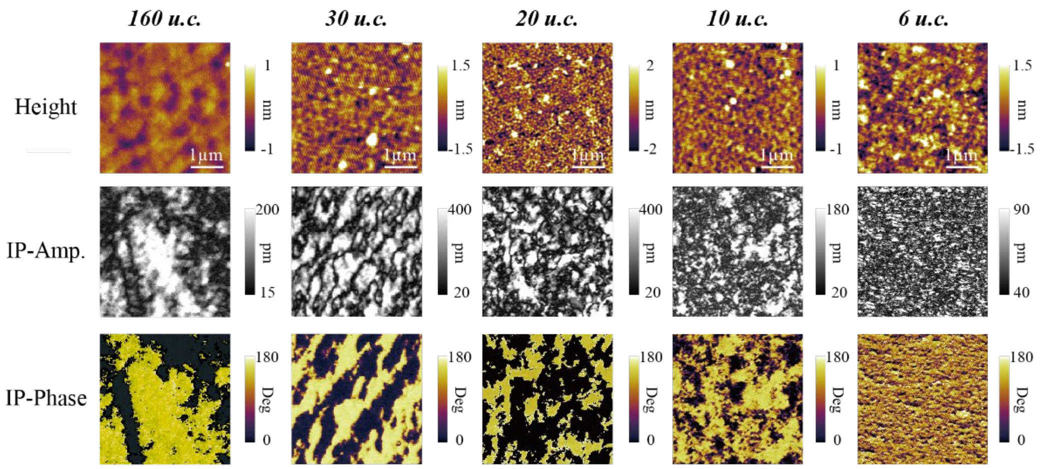

Supplementary Fig. 15 | Thickness dependence of in-plane domain structures. In-plane domain structures acquired by LPFM for freestanding BTO membranes of various thicknesses, demonstrating the overall tendency of increased domain wall density with decreased film thickness.

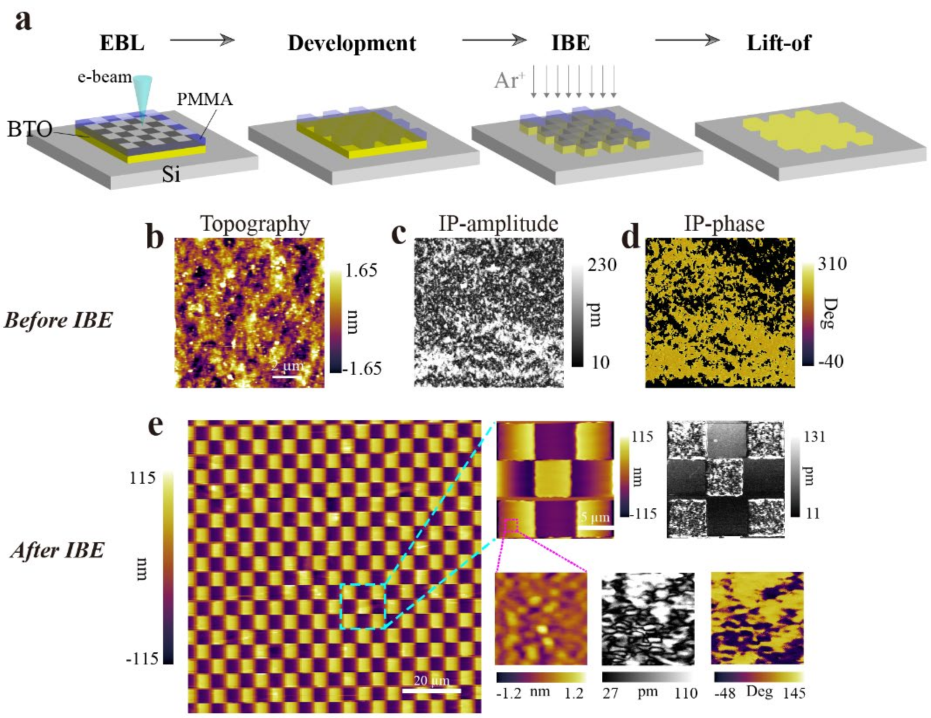

Supplementary Fig. 16 | LPFM results before and after ion-beam lithography on 20 u.c. BTO/Si. a, Schematic illustration for fabricating square-patterned BTO on Si, through electron-beam lithography (EBL) and Ar^+ ion-beam lithography (IBE). **b-d,** LPFM results of pristine BTO/Si. **e,** LPFM results of BTO/Si after ion-beam lithography.

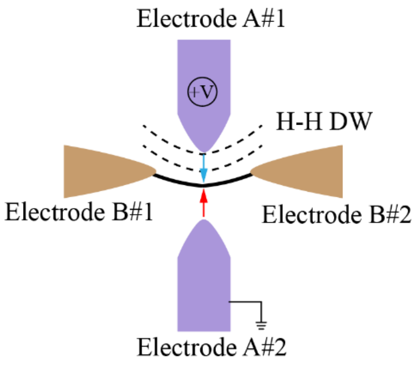

Supplementary Fig. 17 | Schematics of a four-terminal model. Four-terminal model may be applicable to BTO/Si prototype, with two terminals (purple) used for ‘writing’ and the other two (brown) for ‘reading’. Arrows represent polarization orientations.

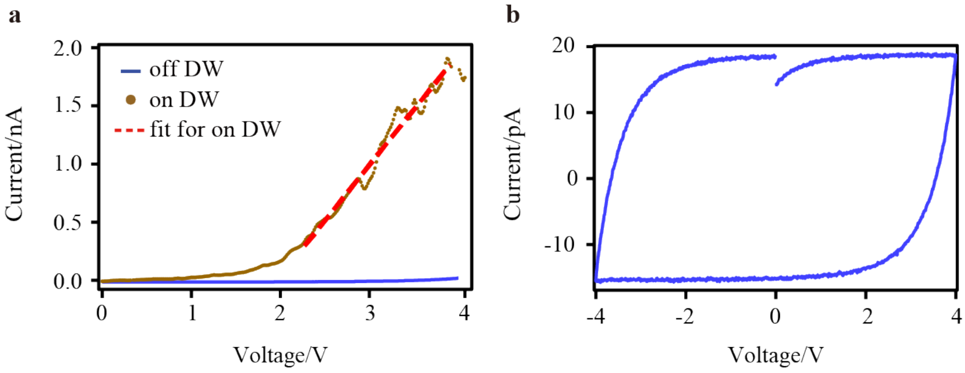

Supplementary Fig. 18 | I-V curves off and on DW. a, I-V curves on and off the conductive DWs shown in Fig. 4b. **b,** 'Off DW' I-V hysteresis.

Supplementary Table 1

Values of parameters used in this work (SI units and T at 300 K)[58].

Parameter	Value	Parameter	Value
α_1(J·m·C^{-2})	-2.7104××10^{7}	c_{44}(J·m^{-3})	5.43×10^{10}
α_{11}(J·m^{5}·C^{-4})	-6.3887×10^{8}	Q_{11}(m^{4}·C^{-2})	0.1104
α_{12}(J·m^{5}·C^{-4})	3.23×10^{8}	Q_{12}(m^{4}·C^{-2})	-0.0452
α_{111}(J·m^{9}·C^{-6})	7.9019×10^{9}	Q_{44}(m^{4}·C^{-2})	0.0578
α_{112}(J·m^{9}·C^{-6})	4.47×10^{9}	G_{11}(N·m^{4}·C^{-2})	3.46×10^{-10}
α_{123}(J·m^{9}·C^{-6})	4.91×10^{9}	G_{12}(N·m^{4}·C^{-2})	0
c_{11}(J·m^{-3})	2.75×10^{11}	G_{44}/G'_{44}(N·m^{4}·C^{-2})	1.73×10^{-11}
c_{12}(J·m^{-3})	1.79×10^{11}	κ_b(1)	50
